# The significance of MUAC z-scores in diagnosing pediatric malnutrition: A scoping review with special emphasis on neurologically disabled children

**DOI:** 10.3389/fped.2023.1081139

**Published:** 2023-03-06

**Authors:** Kürşad Aydın, Buket Dalgıç, Aydan Kansu, Hasan Özen, Mukadder Ayşe Selimoğlu, Hasan Tekgül, Bülent Ünay, Aysel Yüce

**Affiliations:** ^1^ Department of Pediatric Neurology, Medipol University Faculty of Medicine, Istanbul, Türkiye; ^2^ Department of Pediatric Gastroenterology, Gazi University Faculty of Medicine, Ankara, Türkiye; ^3^ Department of Pediatric Gastroenterology, Ankara University Faculty of Medicine, Ankara, Türkiye; ^4^ Department of Pediatric Gastroenterology, Hacettepe University Faculty of Medicine, Ankara, Türkiye; ^5^ Department of Pediatric Gastroenterology, Memorial Atasehir and Bahcelievler Hospitals, Istanbul, Türkiye; ^6^ Department of Pediatric Neurology, Ege University Faculty of Medicine, Izmir, Türkiye; ^7^ Department of Pediatric Neurology, Gulhane Faculty of Medicine, Ankara, Türkiye

**Keywords:** pediatric malnutrition, diagnosis, anthropometry, MUAC z-score, MUAC z-score tape, neurological disability

## Abstract

This review by a panel of pediatric gastroenterology-hepatology-nutrition and pediatric neurology experts aimed to address the significance of mid-upper arm circumference (MUAC) assessment in diagnosis of pediatric malnutrition. Specifically, the potential utility of recently developed MUAC z-score tape in clinical practice for larger patient populations was addressed including the neurologically disabled children. In accordance with the evidence-based data, four statements were identified by the participating experts on the utility of MUAC z-score tape, including (1) MUAC z-scores correlate with body mass index (BMI) and weight for height/length (WFH/l) z-scores in diagnosing malnutrition; (2) MUAC *z*-score tape offers a higher sensitivity to diagnose the mild and moderate malnutrition and better ability to track the changes in nutritional status over time than the other single datapoint measurements; (3) Using single-step MUAC *z*-score tape in children with cerebral palsy (CP) seems to provide more reliable data on anthropometry; and (4) The clinical value of the tool in classifying secondary malnutrition in CP should be investigated in large-scale populations. In conclusion, enabling single-step estimation of nutritional status in a large-scale pediatric population regardless of age and within a wide range of weight, without formal training or the need for ancillary reference charts and calculators, MUAC *z*-tape offers a favorable tool for easier and earlier diagnosis of pediatric malnutrition. Nonetheless, further implementation of MUAC *z*-score screening in larger-scale and/or special populations is necessary to justify its utility in relation to other primary anthropometric indicators in diagnosis of malnutrition as well as in treatment monitoring in the community and hospital setting.

## 1. Introduction

Pediatric malnutrition remains to be a highly prevalent problem worldwide, whereas it is frequently underdiagnosed or underestimated in clinical practice due to diagnostic challenges (1–4). Malnutrition can cause important consequences not only in childhood but also in adulthood. The younger and more severely malnourished the person is, the worse the consequences (5–11). Hence, preventing all grades of malnutrition through timely recognition and treatment of malnutrition risk/malnutrition is associated with improved child survival and a healthier life (1–3, 10, 12).

Weight-for-length (WFL; in infants and children up to 23 months of age), weight-for-height (WFH; in children aged ≥24 months), mid-upper arm circumference (MUAC; in children aged ≥2 months), and body mass index (BMI; in those aged 2 to 19 years) are the most widely used anthropometric measurements in diagnosis of pediatric malnutrition (12–15). Recently, MUAC *z*-score tape was developed to further facilitate the diagnosis of primary malnutrition, which enables single-step assessment of nutritional status (as defined by *z*-score) without using ancillary reference charts and calculators, and for a larger number of children regardless of the any age and across a wide weight range (16–22).

Diagnosis of malnutrition is considerable challenging in neurologically disabled children, children with cerebral palsy (CP) in particular, due to difficulty in obtaining reliable anthropometric measurements in case of joint contractures (23–25). These children are also at risk of misestimation of the nutritional status, and this risk remains unperturbed independently of the tool used, if specific charts for this special population are not available or not used (24, 26–28). In this regard, the MUAC *z*-score tape may represent a favorable assessment tool to obtain a more accurate anthropometric profile in children with CP and to improve nutritional rehabilitation practice in this setting.

This scoping review aimed to address the significance of MUAC among other primary anthropometric indicators in diagnosis of pediatric malnutrition and the potential utility of recently developed MUAC *z*-score tape in the clinical practice in diagnosis of malnutrition for larger-scale and/or special patient populations including the neurologically disabled children.

## 2. Methods

This scoping review was prepared by an expert panel of pediatric gastroenterology-hepatology-nutrition and pediatric neurology specialists. The panel critically analyzed recommendations from international guidelines and consensus statements, systematic reviews, results of randomized control trials, population-based studies, prospective longitudinal cohort studies, multicenter cross-sectional studies and case reports focusing on diagnosis of malnutrition and anthropometric assessments with use of MUAC *z*-scores in particular, in the community and hospital setting. The significance of MUAC in diagnosis of pediatric malnutrition and the potential utility of recently developed MUAC *z*-score tape in the clinical practice for special patient populations including the neurologically disabled children were discussed, as supported by scientific evidence and expert clinical opinion.

The main topics addressed in this paper are a) pediatric malnutrition (definition, classification, prevalence, primary anthropometric diagnostic indicators), b) the significance of MUAC as a single datapoint anthropometric measurement (criteria for an ideal malnutrition screening tool, role of MUAC in nutrition programming and mortality prediction, MUAC *z*-score in relation to WFH/l and BMI *z*-scores), c) assessment of MUAC *z*-score (traditional MUAC tape vs. MUAC *z*-score tape), and d) potential utility of MUAC *z*-score tape in neurologically disabled children.

## 3. Pediatric malnutrition

### 3.1. Definition, classification and prevalence of pediatric malnutrition

American Society for Parenteral and Enteral Nutrition (ASPEN) defines pediatric malnutrition (undernutrition) as an imbalance between nutritional need and intake, resulting in cumulative deficits of energy, protein, or micronutrients with potential adverse effects on growth, development, and other related outcomes (14). The classification of malnutrition is based on its etiology (primary, secondary), duration (acute, chronic), anthropometrics (stunting, wasting and underweight) and severity (mild, moderate, severe) (14).

Height or length for age is a criterion for assessing stunting secondary to chronic malnutrition, while WFH/l is used for assessing wasting due to acute malnutrition. Weight for age (WFA) is a growth indicator that relates weight to age and measures underweight, which may be low in acute and/or chronic malnutrition (29). The infants and children below age 5 are at greater risk of malnutrition due to accelerated growth and brain development or inappropriately introduced complementary foods (too early, too late, low nutrient density and micronutrient bioavailability) (1, 4, 12).

Although there has been a decreasing trend since 2000, stunting and wasting are still seen in 22% and 6.7% of children <5 years of age globally in 2020, respectively (30). Malnutrition, particularly wasting, increases risk of death, and is associated about half of all under-5 deaths (14, 30). In Turkey, stunting is the most common form of malnutrition with considerably higher prevalence of stunting (6.1% in boys and 5.8% in girls) than underweight (1.3% and 1.7%, respectively) and wasting (1.7% and 1.6%, respectively) in children below five years (31).

Non-illness related (primary) malnutrition is a multifactorial condition with potential contribution of poor maternal nutrition, low birth weight, insufficient breastfeeding or complementary feeding, poverty, lack of adequate food, recurrent infections and environmental enteropathy. Illness-related (secondary) malnutrition occurs in the presence of an underlying disease with adverse effects on growth, particularly the neurological diseases like CP, chronic infectious diseases, cystic fibrosis, congenital cardiovascular diseases, primary immunodeficiencies, malignancy and certain gastrointestinal and liver diseases (4, 14). While several factors related to underlying disease may contribute to malnutrition, malnutrition itself also leads to poor response to treatment of the underlying disease if not recognized or left untreated (4, 12, 14).

### 3.2. Diagnosis of pediatric malnutrition (undernutrition): A shift from the use of percentiles to z-scores with single datapoint anthropometric measurement

Although ASPEN's definition of pediatric malnutrition comprises several aspects of poor health outcomes in a malnourished child, there remain challenges in everyday clinical practice in its implementation (12, 14, 15). In an attempt to standardize the approach to diagnose malnutrition in children and to improve overall nutrition assessment and consistency, Academy of Nutrition and Dietetics (AND)/ASPEN reached a consensus in 2014 with regard to the use of z-scores for WFH/l, BMI for age, L/HFA, or MUAC when a single data point is available (15).

The WFH/l *z*-scores and BMI z-scores are more commonly used in health care settings, since these measurements necessitate formal training and access to special equipment (i.e., scale, stadiometer, anthropometric caliper and calculation). However, the MUAC, as measured in mm using a measuring tape that also displays the *z*-score, gives the severity of malnutrition with a single measurement, and can be used in any condition. Accordingly, it is considered an easier method to be adopted by front-line health workers for screening of acute malnutrition in a community setting (12, 21, 32–34).

The global evidence indicates higher sensitivity of MUAC than WFH z-score in identifying children at higher risk for severe acute malnutrition and in predicting mortality (19, 35–37). In addition, the updated WHO and UNICEF guidelines on a standardized cut-off enabled MUAC to become a widely used and successful diagnostic tool for screening children in medically underserved and resource restricted settings (38). However, the cut-offs for severe and moderate acute malnutrition, which are well-established for WFH and BMI *z*-scores, are considered to be much less consistent for traditional MUAC tapes (16, 21). Hence, reference growth curves have been developed for age-specific MUAC *z*-scores by several groups (17–19), while MUAC z-scores defined for children aged 2 months to 18 years in the United States. cohorts in 2017 has extended the utility of this single datapoint of arm anthropometry in diagnosis and classification of malnutrition (19).

In the recent AND/ASPEN consensus statement, MUAC was specifically listed amongst the independent indicators for diagnosing pediatric malnutrition in children 6 to 59 months of age (15). The use of single datapoint of anthropometry based on WFH/l, BMI or MUAC *z*- scores (−1 to −1.9: mild malnutrition, −2.0 to −2.9: moderate malnutrition and ≥ −3: severe malnutrition) is considered for diagnosis of malnutrition when a child has only a single datapoint (Table 1) (15).

**Table 1:** Single datapoint of anthropometry and additional supportive criteria for diagnosis and classification of pediatric malnutrition (15).

	Mild malnutrition	Moderate malnutrition	Severe malnutrition
**Single datapoint of anthropometry**			
WFH/l *z*-score	−1 to −1.9	−2 to −2.9	−3 or greater
BMI for age *z*-score			
MUAC *z*-score			
**Additional supportive criteria**			
Weight gain velocity (<2 y)	<75% of the norm	<50% of the norm	<25% of the norm
Weight loss (2 to 20 y)	5%	7.5%	10%
Deceleration in WFH/l *z*-score	Decline of 1 *z*-score	Decline of 2 *z*-score	Decline of 3 *z*-score
Inadequate nutrient intake (% of estimated energy/protein need)	51% to 75%	26% to 50%	≤25%

WFH/l: weight for height/length; BMI: body mass index; MUAC: mid-upper arm circumference.

When a child presents with historical medical information and two or more data points are available for use as criteria, additional criteria including weight gain velocity (<2 y), weight loss (2 to 20 y), deceleration in WFH/l *z*-score and inadequate nutrient intake can also be used to support malnutrition diagnosis (Table 1) (15).

## 4. The significance of MUAC as a single datapoint measurement

### 4.1. MUAC as an ideal malnutrition assessment tool

The ideal method for malnutrition screening and case detection in the community setting is considered to be simple, acceptable, low cost, precise, accurate, sensitive, specific and predictive and to be both objective and quantitative allowing for completeness of coverage (39). In addition, for detecting cases of malnutrition in early childhood, indicators are also suggested to be age-free, given the challenges in estimating accurate age in the context of severe malnutrition-based treatment programs (39).

The subjective clinical assessment (i.e., visible severe wasting) is considered to perform poorer than any anthropometry-based method, while WFH/l-based case-detection methods are considered to perform worse than MUAC, especially in certain circumstances (39, 40). Hence, MUAC, based on the currently available evidence, is considered to be the best case-detection method for pediatric malnutrition in terms of easiness, age independence, precision, accuracy, sensitivity, specificity and lack of special equipment, formal training requirement or calculation (Table 2) (39, 40).

(a) **Simplicity:** MUAC was reported to be faster and easier to perform screening in the community setting than other indicators including WFA, HFA and WFH (41) and to reveal immediate classifications of malnutrition by using a readily understandable “traffic light” system with colors (39).
(b) **Acceptability, precision and accuracy:** MUAC offers a higher acceptability and thereby an improved precision and accuracy of measurements in younger children who are agitated during weight and height measurements (39).
(c) **Age independence in mortality risk prediction:** MUAC and WFH are known to be “relatively independent” and “independent” of age between 1 and 5 years of age, respectively. However, while the power of WFH in predicting mortality may change with age, the mortality-predicting power of MUAC is independent of age and is valid even in children below age 1 (39, 42).

**Table 2:** Key properties of WFA, WFH/l and MUAC *z*-score tools (39, 40, 43, 51, 55, 56).

	WFA	WFH/l	MUAC
**Property**			
Simplicity	No	No	Yes
Acceptability	No	No	Yes
Objectivity	No	Yes	Yes
Precision (reproducibility)	Yes	No	Yes
Reliability in case of difficulty in obtaining anthropometric measurements	No	No	Yes
Accuracy in the presence of edema, ascites or organomegaly	No	No	Yes
Sensitivity for diagnosing mild-to-moderate malnutrition	Low	Low	High
Sensitivity of serial measurements to track nutritional status over time	Low	Low	High
Mortality prediction	Weak and possibly age-dependent	Weak and possibly age-dependent	Strong and age-independent
Formal training	Recommended	Recommended	Not required
Equipment	Scale: standing, sitting, hoist; stadiometer; measuring tape; anthropometric caliper	Only measuring tape

WFA: weight-for-age; WFH/l: weight for height/length; MUAC: mid-upper arm circumference.

### 4.2. MUAC in nutrition programming (screening, admission, monitoring recovery) and mortality prediction

Use of WFH *z*-score alone or in combination with MUAC has no superiority over MUAC alone in identifying children at risk of mortality. Growing evidence indicates that weight gain and MUAC gain follow each other and similarly response to the treatment (43–46). Thus, MUAC is increasingly recognized as a stand-alone favorable tool that can be used for every phase of the entire nutrition programming (i.e., screening, hospital admission, disease monitoring and time of discharge) (46).

Many studies indicated MUAC as an independent tool for diagnosing malnutrition in children aged 6-59 months, which is found to be correlated to BMI but to be a more sensitive prognostic indicator for mortality than WFH in children and to be more sensitive to changes in muscle and fat mass than BMI in adults (14, 43, 47–51).

In a systematic review in 2013, while MUAC was concluded to offer an appropriate stand-alone criterion for hospital admission and discharge due to severe acute malnutrition, the MUAC cutoffs were reported to range from 110 to 130 mm for children aged 6-59 months (52). Although controversial, the reliability of MUAC in diagnosing severe acute malnutrition among infants at ≤6 months age is suggested to be low due to lack of data on its inter-observer reliability (53). In this age group, MUAC is also considered to be further tested in terms of predicting malnutrition-associated adverse outcomes (i.e., risk of infections, morbidity and mortality) (54).

### 4.3. MUAC z-score in relation to WFH/l z-score and BMI z-score

When compared to WFH/l and BMI z-scores, MUAC z-scores are considered to be more sensitive to diagnose mild-to-moderate malnutrition and to track the change in nutritional status overtime, and also to provide more reliable data in the presence of ascites or edema, being not affected by fluid shifts or hydration status (Table 2) (43, 51, 55, 56).

In a recent study assessing the correlation of the AND/ASPEN single datapoint indicators in diagnosis of pediatric malnutrition in a small cohort of children with cystic fibrosis; the correlation between MUAC and WFH/l- BMI *z*-scores were reported to be high in diagnosing malnutrition but not in categorizing malnutrition (55). When compared to BMI/WFL *z*-scores, MUAC *z*-scores identified more children in the undernourished category and diagnosed mild-to-moderate malnutrition with a higher sensitivity (55). These findings seem notable given that mild-to-moderate malnutrition has a more challenging diagnosis, despite it comprises the majority of malnourished cases and accounts for majority of malnutrition-related deaths (57, 58). In addition, MUAC *z*-scores but not the corresponding BMI/WFL z-scores showed a significant change when tracked over time, emphasizing the likelihood of serial measurements of MUAC to pick up smaller improvements in lean body mass than BMI or weight change and to be a better method for assessing nutritional status overtime (55). Hence, the favorable utility of MUAC *z*-scores particularly in the setting of accompanying dehydration or edema is emphasized since it is not affected by fluid shifts or hydration status unlike to weight or BMI z-scores (5, 56).

MUAC measurements are suggested to be appropriate particularly in the presence of conditions likely to affect weight (i.e., lower-extremity edema, organomegaly or steroid usage), as weight trends *per se* are not reliable due to fluid status (15). Moreover, serial MUAC z-score measurements are considered to offer more reliable data on body composition monitoring, using the child as his/her own control (15, 43, 51).

In another study, MUAC z-scores were evaluated with respect to the z-scores for conventional indicators (WFL and BMI), using *λ*, *μ*, and *σ* values derived from Centers for Disease Control and Prevention (CDC) reference data in a large cohort of children in United States. practice settings (18). The authors reported that MUAC *z*-scores were significantly correlated with BMI and WFL *z*-scores (18). Also, MUAC *z*-scores were found to span a narrower range of values, since they are slightly smaller (in overweight/obese population) or larger (in severely malnourished children) than the corresponding BMI or WFL *z*-scores (18). Hence, *z*-score ranges used to define the stages of malnutrition may not be the same for all indicators, emphasizing that the different anthropometric indices require index-specific interpretation (18, 41, 59).

While MUAC z-score has become included amongst the indicators for pediatric malnutrition, given that limited experience with regard to implementation of MUAC z-score in clinical practice, further efforts should be made to improve use of MUAC in larger-scale and/or special populations (12, 14, 15, 20).

## 5. Assessment of MUAC z-score: traditional vs. z-score tape

The traditional MUAC tape has been a preferred tool due to its feasibility at community level in diagnosing malnutrition in children aged 6–59 months, as associated with high sensitivity and specificity for global acute malnutrition (defined by a MUAC <125 mm, > 90% and >80%, respectively) and for severe acute malnutrition (defined by a MUAC <115 mm, > 73% and >98%, respectively) (36, 60). Nonetheless, while evidence suggests MUAC as a better predictor of mortality as compared to WFH/l *z*-score, some authors considered that assessing the immediate mortality risk should not be the sole purpose of diagnosing acute malnutrition, and using MUAC-alone will underestimate the prevalence of severe acute malnutrition at the community level given that both WFH/l *z*-score and MUAC are known to identify different sets of children (36).

Given the current recommendations regarding a shift from the use of percentiles to z scores, availability of an accurate and dependable system to calculate these values is of critical importance (12). The normative data for a matched reference population for MUAC *z*-scores are available for children aged 2 months to 18 years (19). However, the traditional MUAC tape is based on a measurement of the MUAC in millimeters and then finding the related *z* score either by using MUAC growth curves or by calculating (manual or automated) *via* the formula based on the MUAC, lambda (L), mu (M), and sigma (S) values defined for the age and gender of the patient (20). In both ways, this is a time-consuming process necessitating use of at least 1 device (a traditional tape measure) and 1 set of references (growth charts or LMS data tables) and challenging for a busy clinician (20). While calculation of MUAC z-scores can also be made using online resources (https://peditools.org/cdcmuac/), this is still a multistep process necessitating presence of the electronic resource (20, 61). In addition, the traditional MUAC tape specifically defines the risk of severe acute malnutrition only in the 6-monthto 5-year age group, and provides only the values which necessitate use of ancillary reference charts for their interpretation (18‒20).

Hence, to overcome these limitations of the traditional MUAC tape, the novel MUAC z-score tape is developed as a prototype device capable of estimating nutritional status (as defined by *z*-score) in a single step process for any child and free of ancillary reference charts and calculators (20).

**Figure 1:**
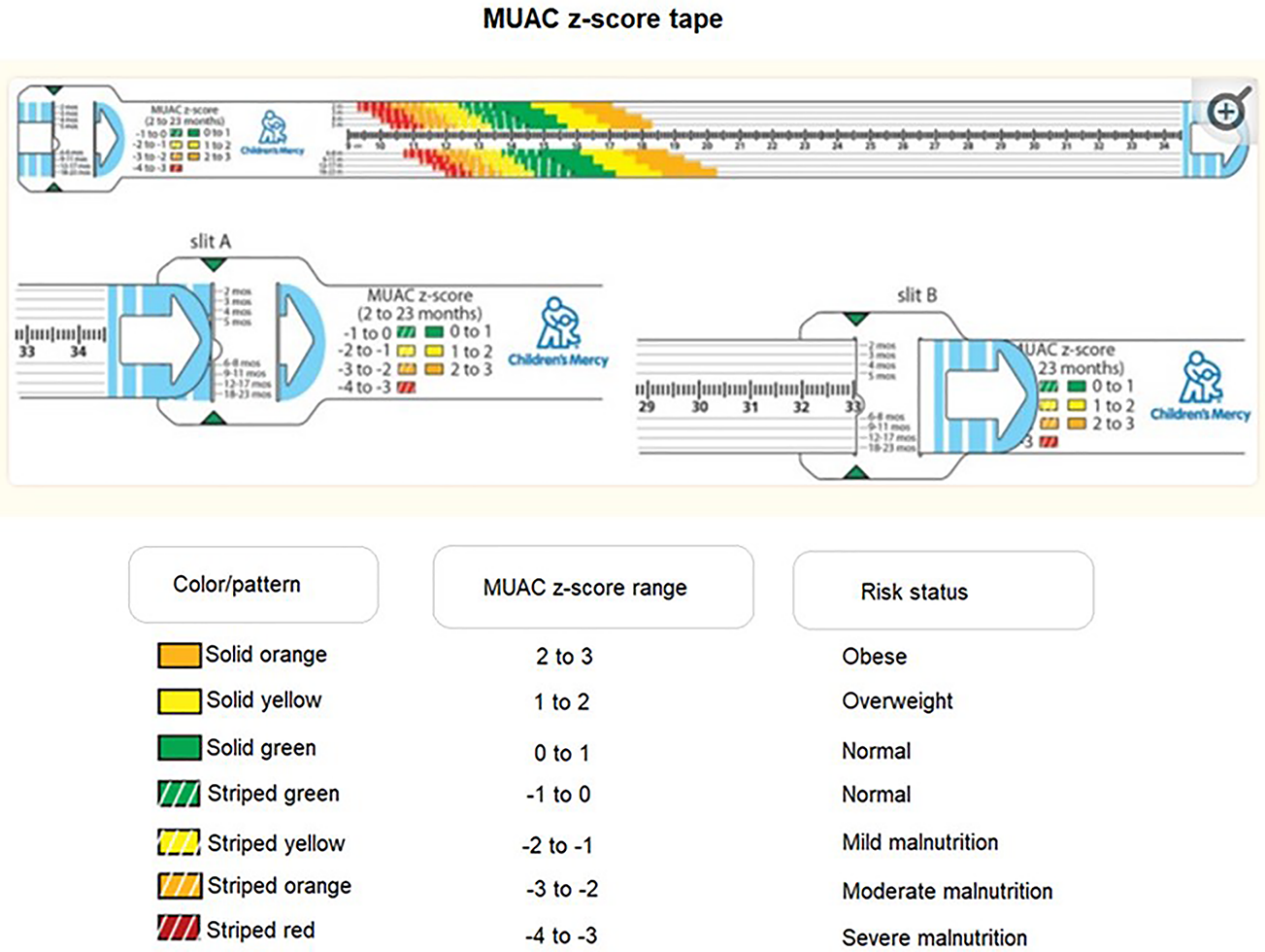
Prototype MUAC z-score tape (adapted from Thaete K et al. User-Informed Medical Device Development: A Case Study for Pediatric Malnutrition Assessment. Glob Pediatr Health. 2019 Jul 15;6:2333794X19861575).

MUAC z-score tape (smaller one for 2-59 months age and larger one for 5-18 years of age) has a traditional measuring scale in metric units which reveals the MUAC, while linear markings with color-coded bands for the selected age-ranges indicates the MUAC *z*-score range (Figure 1) (20).

Circumferential measurements of the upper arm are made at the midpoint between the acromion and olecranon which can be identified using the ruler on the device. Before the measurement, the tail end is threaded into slit “A” and back out through slit “B” to create a loop. At midpoint positioning, the tail end of the tape is pulled enough not to compress the skin. The user identifies the color band corresponding to the age of the patient and records the z-score range (Figure 1) (20).

In a study on the usability and performance characteristics of MUAC z-score tape in a real-world clinical setting (20), perceptions of overall ease of use, efficiency, convenience and acceptability of the device were reported to significantly increase with increasing user experience, and the device was considered preferable to the conventional methods of obtaining MUAC z-score (20).

In another study on the effectiveness of a novel MUAC *z*-score tape to identify children over age 5 who would benefit from nutritional rehabilitation, a total of 818 MUAC *z*-score tape measurements obtained by 112 community health volunteers were assessed (21). MUAC *z*-scores were reported to identify 87.1% (27/31) of the malnourished (severe or moderate) children as determined by the BMI *z*-score as well as with an additional six children not classified as such by the BMI *z*-score (21). Volunteers reported moderate rates of interpreting the nutritional risk using the tape, and 62.5% considered the tape easy to use (21). The authors suggested that with more in-depth training, the MUAC *z*-score tape is a practical, economic and low-burden alternative for community-level screening, enabling more convenient measurement tool than conventional method which requires calculating BMI, converting the BMI to an age- and gender-relevant z-score, and assigning the related nutritional risk (21). Nonetheless, given that only about half of the children with severe acute malnutrition were identified by both methods, the disagreement is considered to be more likely for more extreme values (21). This supports the previous observation in United States. children indicated that z-score thresholds may require refinement for an improved concordance (18).

Although the anthropometric *z*-scores used in diagnosis and classification of malnutrition, specifically BMI, WFL and MUAC *z*-scores, are not exactly concordant, the thresholds proposed for classification are identical (62). Indeed, the sensitivity of proposed MUAC *z*-score thresholds was reported to systematically decrease with increasing severity of malnutrition, emphasizing the need for optimization to decrease the risk of misclassification and for further complementary data from longitudinal studies (62).

Accordingly, while the MUAC *z*-score tape seems to meet the criteria of a desirable (i.e., inexpensive, noninvasive, easy to implement) pediatric malnutrition screening tool (20, 63), it has yet to be evaluated in larger populations by increasing the accessibility to device not only for community health workers and volunteers but also in the hospital setting (21, 32).

## 6. Utility of MUAC z-score tape in neurologically disabled children

A significant proportion of neurologically disabled children, particularly those with severe and longer-term gross motor impairment and oropharyngeal dysfunction, are considered to be at high risk of poor nutritional status (23–25, 64, 65). The primary neurological insult in these children adversely affects not only the physical and mental capacity but also the enteric neural pathways and compromise the adequate nutrient intake by causing, vomiting, dysphagia swallowing deficits, gastroesophageal reflux, aspiration and constipation. Hence, functional feeding disorders and gastrointestinal dysfunction play a major role in the onset of malnutrition in neurologically disabled children (24, 65–67). Malnutrition itself has also been associated with further risk of poor health outcomes, respiratory and cardiac dysfunction and mortality (64, 65, 68, 69). Accordingly, screening for nutritional status and early recognition and management of malnutrition is considered essential for the optimal care in neurologically disabled children, particularly in those with CP, known as the most prevalent cause of motor disability in children (23, 24, 27, 64, 65).

However, the challenges inherent in anthropometry in children with CP due to joint contractures, muscle atrophy and movement disorders complicate the nutritional assessment by decreasing the likelihood of obtaining reliable measurements of basic anthropometric data (24, 26–28). The consequent risk of incorrect interpretation and analysis of data prevents the correct identification of children at risk of malnutrition (24, 26–28). Besides, evaluation of these children with reference standards for healthy children is also problematic given the high prevalence of linear growth retardation and/or an altered body composition in this population, and there remains a risk of misestimating the nutritional status with any tool, unless the specific charts for this population are used (26, 28). Using the reference of the National Center for Health Statistics of the CDC (NCHS/CDC) in children with CP was reported to be associated with excessively increased (80%) rate of probable malnutrition (70). Notably, in a study from Turkey in 1,108 children with CP, the prevalence of malnutrition was reported to be 57.2% based on physicians' clinical judgment, whereas to be 94.3% (3rd degree in 86.7%) and 91.3% (severe in 88.3%) according to Gomez classification of WFA percentiles and Waterlow classification of HFA percentiles, respectively (24). The authors indicated high prevalence of malnutrition in children with CP, especially in those with higher levels of gross motor dysfunction, while they also emphasized that use of growth charts for general pediatric population for anthropometric assessment may lead to overestimation of malnutrition in children with CP (23). Likewise, other studies also reported that use of anthropometric charts for healthy children in assessing the nutritional status in children with CP reveals malnutrition rates ranging between 40% and 90%, depending on the study population and anthropometric tool (23, 71–73).

Accordingly, given the weak correlation between standard growth charts and specific references for CP, particularly in case of higher severity of motor impairment, using curves with special reference standards for CP population is considered important in nutritional screening of children with CP, while the existing CP growth charts are not recommended in clinical practice (24, 26, 28, 40, 64, 72, 74, 75). Therefore, there is still room for improvement in nutritional rehabilitation practice among children with CP in terms of assessment tools that enable developing more realistic nutritional goals in accordance with the accurately identified anthropometric profile (24, 40, 64, 75).

The nutritional assessment of neurologically disabled children is based on low WFH/l, low TSFT, and low mid-upper arm fat or muscle area according to European Society for Pediatric Gastroenterology, Hepatology and Nutrition (ESPGHAN) guidelines, which is time-consuming and requires trained staff (40, 74). In children older than 2 years with CP, obtaining weight by direct measurement becomes more difficult due to lack of balance and the motor compromise preventing them to stay still on a regular scale, particularly in low- and middle-income countries with limited access to wheelchair adapted scales or even regular scales (76).

MUAC, a segmental measure indirectly assessing growth and changes in caloric and protein intake, uses the fat, bone, and muscle area of the arm as an indirect measure to evaluate body weight (24, 76, 77). Accordingly, using circumferences, primarily MUAC, in combination with other methods has consistently been recommended in children with CP (40).

Notably, in a past study on development of an equation to estimate weight in children with CP, MUAC was reported to be significantly correlated with the weight in males (*q* = 0.87, 0.83–0.90, R2 = 0.81, *p* < 0.001) and in females (*q* = 0.88, 0.83–0.91, R2 = 0.79, *p* < 0.001), while both weight (mean 31.9 vs. 25.6 kg, *p* < 0.001) and MUAC (mean 20.8 vs. 19.0 cm, *p* < 0.001) values were significantly higher in children with GMFCS level I-III than in those with GMFCS level IV-V (76).The authors indicated the age and GMFCS level to be the variables that best adjust the results of the equation to predict weight (76). Hence, combined use of MUAC, age, and GMFCS level is suggested to accurately predict weight in children and adolescents with CP from 2 to 19 years of age (76). Online weight calculator programs (http://inicye.webs.fcm.unc.edu.ar/weight-calculator-cp/) for the weight estimation in children with CP are also based on entry of age, GMFCS level and MUAC data.

In a study with 68 neurologically disabled children (aged 1-17 years, 83.8% with CP), assessment of anthropometry (weight, height, triceps skinfold thickness [TSFT], MUAC) expressed as z-scores revealed the presence of poor nutritional status in 49.5% of patients overall (66). Although MUAC can estimate muscle mass in standard equations, since its use has not been validated in CP, it can underestimate muscle mass in children with such disabilities (65, 78). The assessments based on the “low MUAC” as compared with those based on “low WFH z-score” were reported to identify severe malnutrition in a considerably lower percentage of children (79, 80). These problematic characteristic of MUAC should be further investigated by comparative studies in larger cohorts of children with CP to clarify whether it represents a sufficiently sensitive tool in CP population.

Single-step MUAC *z*-score tape in children with CP seems to provide a faster, easier and more reliable anthropometric assessment tool given the challenges in obtaining reliable measurements of weight, height, and BMI in these children with joint contractures, muscle atrophy, and movement disorders. In addition, serial MUAC *z*-scores are considered to better reflect the change in nutritional status overtime than BMI or weight change (55). Along with the likelihood of accurately estimating weight based on MUAC, age, and GMFCS level in children with CP (76), these features seem to indicate potential utility of MUAC *z*-score tape in diagnosis of malnutrition as well as monitoring the treatment outcomes in children with CP. However, there are areas that mandate further research such as the adaptability and/or consistency of MUAC *z*-scores defined on the tape in classifying malnutrition in children with CP as well as the identification of most appropriate additional criteria supporting the MUAC *z*-score-based diagnosis in this population.

The specific growth charts available for children with CP are also questionable, given that CP is commonly diagnosed after 1 year of age, while the earlier diagnosis of malnutrition and provision of nutritional support is particularly important within the first 2 years of life. Hence, growth charts specific for children with CP will be clinically meaningful and useful only if nutritional support is provided in the earliest possible time period in malnourished children regardless of the time of CP diagnosis. Besides, given the high prevalence of malnutrition in CP which challenges the determination of normal reference values in this population, use of normal reference data with targets specified for CP population seems more reasonable.

## 7. Conclusion

In conclusion, this review by pediatric gastroenterology-hepatology-nutrition and pediatric neurology experts emphasizes that MUAC *z*-scores correlate with WFH/l and BMI *z*-scores in diagnosing malnutrition, while offer a higher sensitivity to diagnose the mild-to-moderate malnutrition, more reliable data in case difficulty in obtaining anthropometric measurements or in the presence of ascites and edema and better ability to track the changes in nutritional status over time than the other single datapoint measurements. These features combined with the further advantages offered by MUAC z-tape (single-step estimation of nutritional status, as defined by *z*-score, for any child, at any age, across a broad weight range, without formal training or the need for ancillary reference charts and calculators) seems to indicate that using MUAC *z*-score tape as an independent single datapoint anthropometry tool may increase the likelihood of diagnosing pediatric malnutrition in larger and/or special populations. The likelihood of accurately estimating weight based on MUAC, age and GMFCS level in children and adolescents with CP and better reflection of the change in nutritional status overtime *via* MUAC *z*-scores than BMI or weight change, the single-step MUAC *z*-score tape seems to be a valuable tool in diagnosing malnutrition and monitoring treatment in children with CP. Accordingly, further implementation of MUAC *z*-score screening in larger-scale and/or special populations is necessary to justify its utility in relation to other primary indicators of single data point anthropometry in diagnosis of malnutrition as well as in treatment monitoring in the community and hospital setting.
